# Novel Scoring Systems to Predict the Need for Oxygenation and ICU Care, and Mortality in Hospitalized COVID-19 Patients: A Risk Stratification Tool

**DOI:** 10.7759/cureus.27459

**Published:** 2022-07-29

**Authors:** Vishakh C Keri, Pankaj Jorwal, Rohit Verma, Piyush Ranjan, Ashish D Upadhyay, Anivita Aggarwal, Radhika Sarda, Kunal Sharma, Shubham Sahni, Chaithra Rajanna

**Affiliations:** 1 Infectious Diseases, All India Institute of Medical Sciences (AIIMS), New Delhi, IND; 2 Medicine, All India Institute of Medical Sciences (AIIMS), New Delhi, IND; 3 Psychiatry, All India Institute of Medical Sciences (AIIMS), New Delhi, IND; 4 Statistics, All India Institute of Medical Sciences (AIIMS), New Delhi, IND

**Keywords:** scoring system, mortality, icu, oxygen, c-reactive protein, neutrophil-lymphocyte ratio, covid-19

## Abstract

Introduction: A rapid surge in cases during the COVID-19 pandemic can overwhelm any healthcare system. It is imperative to triage patients who would require oxygen and ICU care, and predict mortality. Specific parameters at admission may help in identifying them.

Methodology: A prospective observational study was undertaken in a COVID-19 ward of a tertiary care center. All baseline clinical and laboratory data were captured. Patients were followed till death or discharge. Univariable and multivariable logistic regression was used to find predictors of the need for oxygen, need for ICU care, and mortality. Objective scoring systems were developed for the same using the predictors.

Results: The study included 209 patients. Disease severity was mild, moderate, and severe in 98 (46.9%), 74 (35.4%), and 37 (17.7%) patients, respectively. The neutrophil-to-lymphocyte ratio (NLR) >4 was a common independent predictor of the need for oxygen (p<0.001), need for ICU transfer (p=0.04), and mortality (p=0.06). Clinical risk scores were developed (10*c-reactive protein (CRP) + 14.8*NLR + 12*urea), (10*aspartate transaminase (AST) + 15.7*NLR + 14.28*CRP), (10*NLR + 10.1*creatinine) which, if ≥14.8, ≥25.7, ≥10.1 predicted need for oxygenation, need for ICU transfer and mortality with a sensitivity and specificity (81.6%, 70%), (73.3%, 75.7%), (61.1%, 75%), respectively.

Conclusion: The NLR, CRP, urea, creatinine, and AST are independent predictors in identifying patients with poor outcomes. An objective scoring system can be used at the bedside for appropriate triaging of patients and utilization of resources.

## Introduction

The coronavirus disease (COVID-19) pandemic has spread globally causing millions of deaths in multiple waves. India recently witnessed a surge of cases in the third wave of COVID-19 dominated by the Omicron variant [1]. The rapidity with which the cases rise during a pandemic challenges the healthcare system to optimize the available infrastructure, manpower, and resources. The spectrum of COVID-19 disease ranges from asymptomatic cases to severe diseases. Although the proportion of patients with severe disease is much lower, the numbers during a pandemic are sufficiently large enough to overwhelm the existing healthcare systems across the world [2].

Adequate allocation of resources requires appropriate triaging of patients and timely identification of patients who are at risk of developing severe disease and hospitalization. There have been multiple studies that have looked at various clinical and laboratory parameters to determine the same. Comorbidities such as diabetes mellitus (DM), hypertension (HTN), and chronic kidney disease (CKD) as well as laboratory parameters such as lymphopenia, elevated inflammatory markers like c-reactive protein (CRP), and D-dimer are observed in severe disease and supposedly predict early mortality. Multiple such predictors are still under investigation. These predictors of progression to severe disease and mortality can also aid in the selection of patients who require early intensive care unit (ICU) care [3-6].

There is a marked difference in the nature and intensity of management of critically ill patients and non-critical patients, so improvisations must be made in monitoring such patients outside the ICU [7]. The improvised monitoring should be able to detect any rapid deteriorations in medical conditions that would require escalation of life-support or ICU care.

The need for oxygenation, ICU, and the risk of mortality are the three key clinical questions faced by policymakers and clinicians while managing hospitalized COVID-19 patients. The present study was conducted to develop a clinical scoring system to answer these questions using the baseline clinical and biochemical predictors. We expect that the scoring system when used in conjunction with the standard of care, would be able to predict early deterioration and critical events in sick patients and guide policymakers to formulate guidelines to optimize the available resources. The study adds to the evolving scientific literature on predictors of severity and mortality among COVID-19 patients with the help of a novel scoring system.

## Materials and methods

This was a prospective observational study conducted in a dedicated COVID-19 ward of a tertiary care hospital in north India from June 2020 to December 2020. The study population included all admitted patients with confirmed severe acute respiratory syndrome coronavirus 2 (SARS-CoV-2) infection (based on clinical features and positive viral nucleic acid test results on nasal/throat swab samples). Patients were classified as having mild (upper respiratory tract symptoms and/or fever without shortness of breath or hypoxia), moderate (respiratory rate ≥ 24/min, breathlessness or saturation of peripheral oxygen (SpO2) between 90% to 94%) or severe disease (respiratory rate >30/min, breathlessness or SpO2 < 90%) as per institutional guidelines. A sample size of convenience was chosen with as many possible patients being included without any exclusion criteria.

The data of patients were documented in a predesigned proforma. The proforma consisted of three sections. The first one included age and sex. The second section included clinical and co-morbidity data. The clinical data were used to classify the severity of the disease along with mortality data and duration of hospital stay. The third section included laboratory data. The basic laboratory investigations including inflammatory markers were done as per the standard of care decided by the treating team; no additional investigations/interventions were done for the study. All the subjects were followed up till death or discharge from the hospital. The study was undertaken after approval (approval no.IEC-293) by the institute ethics committee of the All India Institute of Medical Sciences (AIIMS), New Delhi.

Statistical analysis was done using Stata 12 software (Stata Corp 2011, College Station, TX, USA). Categorical variables are presented as frequency and percentages. Continuous data are represented as mean/standard deviation or median/interquartile range (IQR). For the purpose of analysis, patients in mild clinical severity were classified as not requiring oxygen and those with moderate and severe disease as requiring oxygen. Patients with severe disease were classified as requiring ICU care. Univariate analysis was done to look for any association between the risk factors and the need for oxygen, ICU care, and mortality. Association was seen using the chi-square/Fischer test for categorical variables. Non-parametric tests including the Mann-Whitney u-test were used to assess the association for continuous variables. Clinical and laboratory correlates with p-value <0.1 in the univariable analysis were included in the multivariable logistic regression analysis. Variables with a p-value <0.1 were considered independent predictors. A predictive score for the need for oxygenation, ICU care, and mortality was derived using coefficients of variables from the regression model. Receiver operating characteristics (ROC) curve analysis was used to develop the cut-off value of the predictive score. Characteristics and performance of score cut-off were reported using sensitivity, and specificity. A p-value <0.05 was considered statistically significant.

## Results

Baseline characteristics of the study population

The study included a cohort of 209 hospitalized patients in the wards of a dedicated COVID-19 care center at the All India Institute of Medical Sciences (AIIMS), New Delhi. There were 146 (69.9%) males and 63 (31.1%) females and the median age of patients was 49 years (35 to 63yrs). This included 98 (46.9%) patients with mild illness, 74 (35.4%) patients with moderate disease, and 37 (17.7%) patients with severe disease at admission. Out of 209 patients, 188 (90%) recovered and were subsequently discharged. The median duration of hospitalization was nine days (six to 16 days). Diabetes mellitus (27.8%), hypertension (25.8%), chronic kidney disease (10.5%), and coronary artery disease (CAD) (10.1%) were the most common comorbidities. Few patients had comorbid conditions of chronic liver disease (CLD) (3.8%), post-renal transplant status (3.8%), chronic obstructive pulmonary disease (COPD) (3.3%), solid organ malignancy (5.3%), hematological malignancy (3.3%), and hypothyroidism (3.8%). Mortality in the study population was 10% (21/209). Seventeen of these deaths happened in patients with severe disease while two deaths each happened in the moderate and mild disease categories. All baseline characteristics of the study population are described in (Table 1)

**Table 1:** Baseline characteristics of the study population The total number of patients for which the values were available has been indicated in the table in brackets next to the individual parameter. COPD: Chronic obstructive pulmonary disease, TLC: Total leukocyte count, NLR: Neutrophil-lymphocyte ratio, AST: Aspartate aminotransferase, ALT: Alanine aminotransferase, ALP: Alkaline phosphatase, CRP: C-reactive protein, IL-6: Interleukin 6, LDH: Lactate dehydrogenase

Parameters (N=209)		n (%) or Median (IQR)
Age (years)		49 (35-63)
Male		146 (69.9)
Severity of disease	Mild	98 (46.9)
	Moderate	74 (35.4)
	Severe	37 (17.7)
Duration of hospital stay (days)		9 (6-16)
Mortality		21 (10.1)
Diabetes mellitus		58 (27.8)
Hypertension		54 (25.8)
Chronic kidney disease		22 (10.5)
Coronary artery disease		21 (10.1)
Chronic liver disease		8 (3.8)
Post-renal transplant		8 (3.8)
COPD		7 (3.3)
Solid organ malignancy		11 (5.3)
Hematological malignancy		7 (3.3)
Hypothyroidism		8 (3.8)
Hemoglobin (g/dl) (191)		11.4 (9.1-13)
TLC (cells/dl) (190)		8050 (5300-12500)
NLR (189)		4 (1.5-8.8)
Platelets (cells/dl) (190)		182000 (130500-262000)
AST (IU/L) (190)		36 (25-52)
ALT (IU/L) (191)		27 (17-52)
Bilirubin (mg/dl) (191)		0.7 (0.6-1.2)
ALP (IU/L) (190)		78 (63-112)
Urea (mg/dl) (189)		36 (26-58)
Creatinine (mg/dl) (188)		0.9 (0.7-1.4)
CRP (mg/L) (85)		2.4 (0.98-9.1)
IL6 (pg/ml) (95)		18.8 (6-49.7)
Ferritin (mg/L) (85)		274.4 (101-550)
D-dimer (mg/L) (30)		0.57 (0.27-1.6)
LDH (IU/L) (38)		291 (227.8-359.3)
Procalcitonin (ng/ml) (34)		0.16 (0.08-0.7)

Predictors and scoring equation to determine the need for oxygenation 

Diabetes mellitus (DM) (p=0.03), hypertension (HTN) (p=0.003), CKD (p=0.004), post-renal transplant patients (p=0.047), hypothyroidism (p=0.02), total leucocyte count (TLC) (p<0.001), neutrophil-lymphocyte ratio (NLR) (p<0.001), aspartate transaminase (AST) (p=0.004), urea (p<0.001), creatinine (p=0.004), c-reactive protein (CRP) (p<0.001), interleukin-6 (IL-6) (p=0.001), lactate dehydrogenase (LDH) (p=0.03), and ferritin (p=0.01) were associated with the need for oxygenation among hospitalized COVID-19 patients. Among these, NLR (p<0.001), CRP (p=0.04) and urea (p=0.07) were independent predictors (Table 2).

**Table 2:** Association between clinical and laboratory parameters with the need for oxygenation, need for ICU, and mortality. COPD: Chronic obstructive pulmonary disease, TLC: Total leukocyte count, NLR: Neutrophil-lymphocyte ratio, AST: Aspartate aminotransferase, ALT: Alanine aminotransferase, ALP: Alkaline phosphatase, CRP: C-reactive protein, IL-6: Interleukin 6, LDH: Lactate dehydrogenase, ULN: Upper limit of normal

Parameters		Mortality		p-value	Oxygenation		p-value	ICU requirement		p-value
		Yes	No		Yes	No		Yes	No	
Age (> 40years)		12 (57.1)	87 (46.3)	0.34	80 (72.1)	58 (59.2)	0.05	26 (70.3)	112 (65.1)	0.55
Male		16 (76.2)	130 (69.2)	0.51	76 (68.5)	70 (71.4)	0.64	28 (75.70	118 (68.6)	0.4
Diabetes mellitus		9 (42.9)	49 (26.1)	0.103	38 (34.2)	20 (20.4)	0.03	15 (40.5)	43 (25)	0.055
Hypertension		11 (52.4)	43 (22.9)	0.003	38 (34.2)	16 (16.3)	0.003	16 (43.2)	38 (22.1)	0.008
Chronic kidney disease		6 (28.6)	16 (8.5)	0.004	18 (16.2)	4 (4.1)	0.004	6 (16.2)	16 (9.3)	0.21
Coronary artery disease		4 (19.1)	17 (9.04)	0.24	13 (11.7)	8 (8.2)	0.39	4 (10.8)	17 (9.9)	0.87
Chronic liver disease		2 (9.5)	6 (3.2)	0.19	5 (4.5)	3 (3.1)	0.59	4 (10.8)	4 (2.3)	0.02
Post-renal transplant		2 (9.5)	6 (3.2)	0.19	7 (6.3)	1 (1.02)	0.047	3 (8.1)	5 (2.9)	0.14
COPD		2 (9.5)	5 (2.7)	0.15	6 (5.4)	1 (1.02)	0.08	3 (8.1)	4 (2.3)	0.08
Solid organ malignancy		2 (9.5)	9 (4.8)	0.31	8 (7.2)	3 (3.1)	0.18	4 (10.8)	7 (4.1)	0.1
Hematological malignancy		1 (4.8)	6 (3.2)	0.53	6 (5.4)	1 (1.02)	0.08	3 (8.1)	4 (2.3)	0.08
Hypothyroidism		0	8 (4.3)	1	1 (0.9)	7 (7.14)	0.02	0	8 (4.7)	0.18
Hemoglobin (< 7g/dl)		5 (27.8)	11 (6.4)	0.01	10 (9.5)	6 (7)	0.53	5 (14.7)	11 (7.01)	0.14
TLC (cells/dl)	< 4000	6 (33.3)	107 (62.2)	0.05	49 (46.7)	64 (75.3)	<0.001	10 (29.4)	103 (66.03)	<0.001
	4000-11000	2 (11.1)	14 (18.1)		7 (6.7)	9 (10.6)		3 (8.8)	13 (8.3)	
	>11000	10 (55.6)	51 (29.7)		49 (46.7)	12 (14.1)		21 (61.8)	40 (25.6)	
NLR (>4)		13 (72.2)	81 (47.4)	0.045	65 (63.1)	29 (33.7)	<0.001	24 (72.7)	70 (44.9)	0.004
Platelets (lakh cells/dl)	<1.5	10 (55.6)	103 (59.9)	0.42	61 (58.1)	52 (61.2)	0.82	17 (50)	96 (61.5)	0.22
	1.5-4.5	8 (44.4)	58 (33.7)		37 (35.2)	29 (34.1)		16 (47.1)	50 (32.1)	
	>4.5	0	11 (6.4)		7 (6.7)	4 (4.7)		1 (2.9)	10 (6.4)	
AST (IU/L) >ULN		5 (27.8)	56 (32.6)	0.68	43 (41)	18 (21.2)	0.004	16 (47.1)	45 (28.9)	0.04
ALT (IU/L) >ULN		4 (22.2)	50 (28.9)	0.78	34 (32.4)	20 (23.3)	0.16	8 (23.5)	46 (29.3)	0.5
Bilirubin (mg/dl) >ULN		5 (27.8)	47 (27.2)	1	30 (28.6)	22 (25.6)	0.64	11 (32.4)	41 (26.1)	0.46
ALP (IU/L) >ULN		4 (22.2)	23 (13.4)	0.3	19 (18.1)	8 (9.4)	0.09	6 (17.7)	21 (13.5)	0.53
Urea (mg/dl) >ULN		16 (88.9)	133 (77.8)	0.37	93 (89.4)	56 (65.9)	<0.001	29 (87.9)	120 (76.9)	0.16
Creatinine (>1.2 mg/dl)		11 (61.1)	44 (25.9)	0.002	16 (18.8)	39 (37.9)	0.004	13 (39.4)	42 (27.1)	0.16
CRP (mg/L) >ULN		4 (57.1)	30 (36.1)	0.27	27 (54)	7 (17.5)	<0.001	11 (68.8)	23 (31.1)	0.005
IL6 (pg/ml) >ULN		6 (85.7)	48 (54.6)	0.11	37 (72.6)	17 (38.6)	0.001	14 (82.4)	40 (51.3)	0.02
Ferritin (mg/L) >ULN		7 (87.5)	38 (45.2)	0.03	31 (60.8)	14 (34.2)	0.01	10 (62.5)	35 (46.1)	0.23
LDH (IU/L) >ULN		3 (75)	26 (76.5)	1	21 (87.5)	8 (57.1)	0.03	8 (88.9)	21 (72.4)	0.31

**Figure 1:**
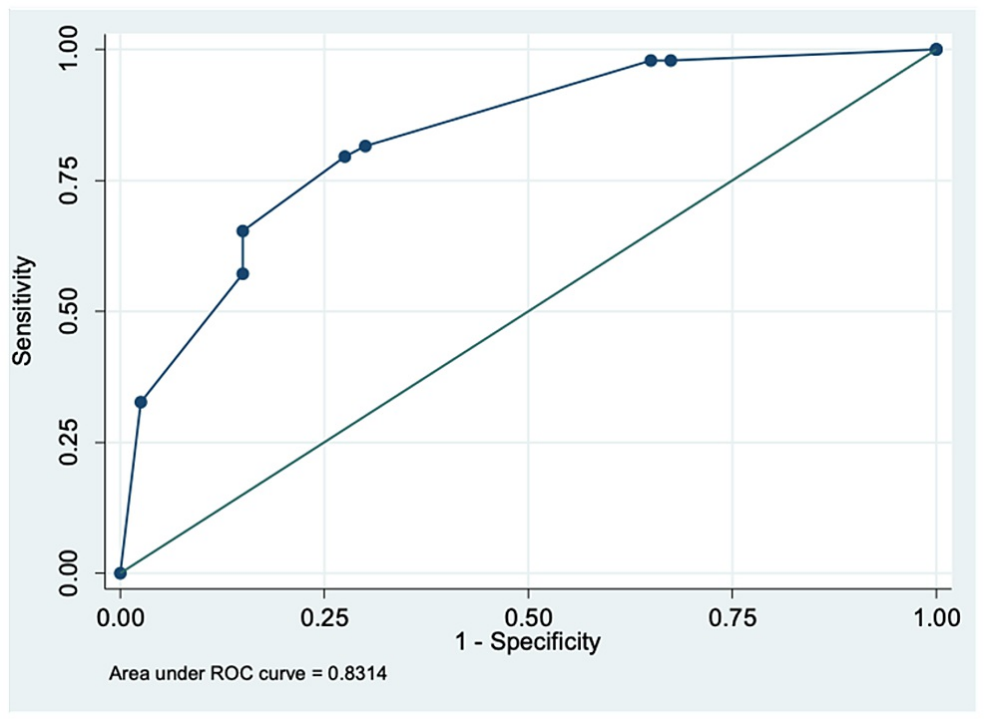
Receiver operator curve (ROC) for scoring system to determine the need for oxygenation

Three variables found significant in the multivariable regression model were used to calculate a predictive score: 10*CRP + 14.8*NLR + 12*Urea (estimated regression coefficient of variable = logistic regression coefficient of variable/logistic regression coefficient of CRP*10) (Table 3). The CRP and urea were assigned a value of “1” if > upper limit of normal (ULN) and “0” if ≤ ULN. The NLR was assigned a value of “1” if >4 and “0” if ≤ 4. Based on ROC curve analysis, the optimal score cut-off for prediction of need for oxygenation in hospitalized COVID-19 patients was found to be ≥14.8 with a sensitivity and specificity of 81.6% and 70%, respectively. The area under the ROC curve was 0.83 (95% confidence interval (CI), 0.75-0.91) (Figure 1).

**Table 3:** Scoring equations to predict the need for oxygenation, need for ICU, and mortality. NLR: Neutrophil-lymphocyte ratio, AST: Aspartate aminotransferase, CRP: C-reactive protein, ULN: Upper limit of normal, AUC: Area under the curve, ROC: Reciever operating characteristics

Scoring equation to predict the need for oxygenation						
Variable	Adjusted odds ratio (95% CI)	p-value	Scoring equation	AUC of ROC	Cut off at ≥14.8	
					Sensitivity	Specificity
NLR (>4)	10.1 (2.639.9)	<0.001	10*CRP + 14.8*NLR + 12*Urea	0.83 (0.75-0.91)	81.6%	70%
CRP (>ULN)	4.2 (1.1-16.3)	0.04				
Urea (ULN)	5.7(0.9-35.9)	0.07				
Scoring equation to predict the need for ICU admission						
Variable	Adjusted odds ratio (95% CI)	p-value	Scoring equation	AUC of ROC	Cut off at ≥25.7	
					Sensitivity	Specificity
NLR (>4)	10.8 (1.2-100.8)	0.04	10*AST+ 15.7*NLR + 14.28*CRP	0.87 (0.78-0.96)	73.3%	75.7%
CRP (>ULN)	8.5 (1.4-52.8)	0.02				
AST (>ULN)	4.5 (0.8-25.1)	0.08				
Scoring equation to predict mortality						
Variable	Adjusted odds ratio (95% CI)	p-value	Scoring equation	AUC of ROC	Cut off at ≥10.1	
					Sensitivity	Specificity
NLR (> 4)	8.1 (0.9-74)	0.06	10*NLR + 10.1*Creatinine	0.73 (0.61-0.85)	61.1%	75%
Creatinine (>ULN)	10.2 (1.8-58.5)	0.009				

Predictors and scoring equation to determine the need for ICU care

Hypertension (HTN) (p=0.008), CLD (p=0.02), TLC (p<0.001), NLR (p=0.004), AST (p=0.04), CRP (p=0.005), IL-6 (p=0.02) were associated with need for ICU care among hospitalized COVID-19 patients. Among these, NLR (p=0.04), CRP (p=0.02) and AST (p=0.08) were independent predictors.

**Figure 2:**
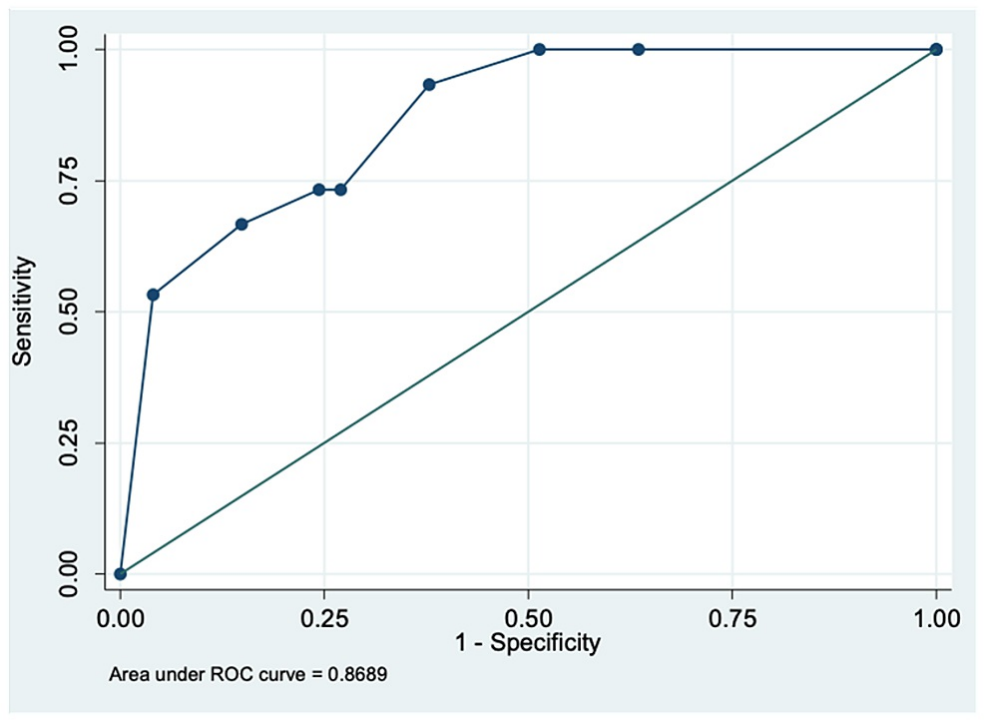
Receiver operator curve (ROC) for scoring system to determine the need for ICU care

Three variables found significant in the multivariable regression model were used to calculate a predictive score: 10*AST+ 15.7*NLR + 14.28*CRP (estimated regression coefficient of variable = logistic regression coefficient of variable/logistic regression coefficient of AST*10). The CRP and AST were assigned a value of “1” if > ULN and “0” if ≤ ULN. The NLR was assigned a value of “1” if >4 and “0” if ≤ 4. Based on the ROC curve analysis, the optimal score cut-off for prediction of need for ICU in hospitalized COVID-19 patients was found to be ≥25.7 with a sensitivity and specificity of 73.3% and 75.7%, respectively. The area under the ROC curve was 0.87 (95% CI, 0.78-0.96) (Figure 2)

Predictors and scoring equation to determine mortality

Hypertension (HTN) (p=0.003), CKD (p=0.004), hemoglobin (p<0.01), NLR (p=0.045), creatinine (p=0.002), and ferritin (p=0.03) were associated with mortality among hospitalized COVID-19 patients. Among these, NLR (p=0.06), and (p=0.009) were independent predictors.

**Figure 3:**
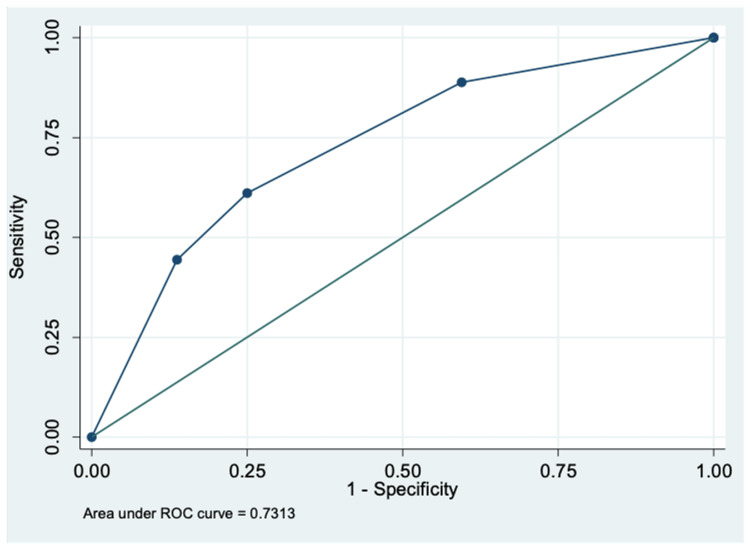
Receiver operator curve (ROC) for scoring system to predict mortality

Two variables found significant in the multivariable regression model were used to calculate a predictive score: 10*NLR + 10.1*creatinine (estimated regression coefficient of variable = logistic regression coefficient of variable/logistic regression coefficient of NLR*10). Creatinine was assigned a value of “1” if > ULN and “0” if ≤ ULN. The NLR was assigned a value of “1” if >4 and “0” if ≤ 4. Based on ROC curve analysis, the optimal score cut-off for prediction of mortality in hospitalized COVID-19 patients was found to be ≥10.1 with a sensitivity and specificity of 61.1% and 75%, respectively. The area under the ROC curve was 0.73 (95% CI, 0.61-0.83) (Figure 3).

## Discussion

A rapid surge in COVID-19 cases disrupted routine healthcare services. Policymakers and clinicians were challenged by oxygen shortages and the lack of ICU beds. A large number of sick patients were managed in the ward setting with limited resources. In such conditions, it becomes imperative to optimally utilize the resources for the patients who could get sick during their hospital stay thus requiring intensive monitoring. Through this study, we have identified predictors and developed a scoring system that can be used by clinicians as a bedside tool to objectively assess individual patients and their need for oxygenation, need for ICU transfer, and the risk of mortality.

The current study involved patients admitted to a COVID-19 ward of an apex COVID care center. Mortality was 10% and almost all of these deaths happened in patients with severe disease, which can be ascribed to delays in presenting to the healthcare system as severe cases constituted up to one-fifth of our patients due to a rapid surge in the number of cases. A study in non-ICU patients in the Cleveland Clinic reported a mortality of 6% with 14% requiring ICU transfer and 14% experiencing an increase in oxygen requirement which was similar to our experience [8]. Mortality as high as 62% has been reported in critically ill patients and up to 81% in those requiring mechanical ventilation [9].

Diabetes mellitus was the most common comorbidity among our patients. All of the comorbidities described as predictors in the available literature were also associated with poor outcomes in our patients on univariable analysis, however, none were independent predictors. A meta-analysis has confirmed that older age, HTN, and DM conferred a significantly increased risk of mortality among patients with COVID-19 [3].

In our analysis, biochemical parameters were the strongest predictors of poor outcomes. A recent meta-analysis also revealed that abnormalities in the biomarkers had higher odds of severe disease and greater risk of mortality [10,11]. Among the biochemical predictors in our study, NLR > 4 was consistently an independent predictor of the need for oxygenation, need for ICU transfer, and mortality. The cut-off of >4 was similar to that seen in multiple other studies [12-15]. A meta-analysis of 13 studies also confirmed the fact that NLR is a good biomarker for predicting disease severity in patients with COVID-19 (area under the curve (AUC)=0.85, sensitivity=0.78, and specificity=0.78), and in predicting mortality as well (AUC=0.90, sensitivity=0.83, and specificity=0.83) [16]. Other parameters including CRP, AST, urea, and creatinine were identified as independent predictors in our study. A meta-analysis of 5350 patients showed that elevated CRP was associated with an increased composite poor outcome (respiratory rate (RR)=1.84) and in the severe COVID-19 (RR=1.41) subgroup. A CRP ⩾10 mg/L has a 51% sensitivity, 88% specificity, and an AUC of 0.84 [17].

The objective scoring system that we developed consistently involved NLR as a component. Among the three scores that we calculated, the equation to predict the need for ICU transfer that included NLR, CRP and AST had the highest discriminatory power with AUC=0.87, followed by the need for oxygenation (AUC=0.83). The equation for mortality had a poorer discriminatory value (AUC=0.73). Multiple studies conducted to determine a scoring system to predict the need for oxygen and mortality have also consistently included NLR and other biomarkers that we have explored [18-23].

Our scoring system is in tune with the scoring systems described in the literature. The unique feature of our study is that we have come up with a composite score by taking all the predictors into account. The scoring system can be utilized to risk-stratify patients even in the current and upcoming pandemics. A similar study done at our institute generated a symptom-based clinical scoring system for diagnosis of COVID-19, however, our study looked into poor prognostic markers in hospitalized COVID-19 patients [24].

Our study had a few limitations. First, most of our variables had a non-normal distribution forcing the use of non-parametric tests to assess the association. Second, the exploration of predictors depends on the number of potential predictors studied. Although we have covered most of the variables we may have missed other clinical predictors which may be further explored. Third, being a ward-based study, the data lack comparison to those patients who get admitted directly to ICU. Fourth, triage thresholds for hospitalization vary from center to center thus limiting the generalizability of our study. And fifth, we did not validate our scoring system, hence further studies may be required for internal and external validation.

## Conclusions

The present study indicates that the presence of NLR > 4, CRP, urea, creatinine, and AST are independent predictors for escalation of care. Clinical risk scores of ≥14.8, ≥25.7, and ≥10.1, predict the need for oxygenation (sesnsitivity=81.6%, specificity=70%), need for ICU transfer (sensitivity=73.3%, specificity=75.7%), and mortality (sensitivity=61.1%, specificity=75%). An objective scoring system that consistently involves NLR can be used by clinicians at the bedside for predicting adverse outcomes. Intensive monitoring and early triaging of such patients can reduce adverse outcomes. 
